# Data on the characterization of Raney nickel powder and Raney-nickel-coated electrodes prepared by atmospheric plasma spraying for alkaline water electrolysis

**DOI:** 10.1016/j.dib.2018.10.167

**Published:** 2018-11-03

**Authors:** Ji-Eun Kim, Ki-Kwang Bae, Chu-Sik Park, Seong-Uk Jeong, Kyeong-Ho Baik, Jong-Won Kim, Young-Ho Kim, Kyoung-Soo Kang, Ki-Bong Lee

**Affiliations:** a Hydrogen Laboratory, Korea Institute of Energy Research, 152 Gajeong-ro, Yuseong-gu, Daejeon 34129, South Korea; b Department of Materials Science and Engineering, Chungnam National University, 99 Daehak-ro, Yuseong-gu, Daejeon 34134, South Korea; c Department of Chemical and Biological Engineering, Korea University, 145 Anam-ro, Seongbuk-gu, Seoul 02841, South Korea; d Department of Chemical Engineering Applied Chemistry, Chungnam National University, 99 Daehak-ro, Yuseong-gu, Daejeon 34134, South Korea

## Abstract

The data presented in this article are related to the research article entitled: “Electrochemical characterization of Raney nickel electrodes prepared by atmospheric plasma spraying for alkaline water electrolysis” (Kim et al., 2018). This article describes the characterization of raw Ni-Al alloy and Raney Ni powders via X-ray diffraction (XRD) and energy dispersive X-ray spectroscopy (EDS), and presents the EDS data of the prepared electrodes.

**Specifications table**

**Table:** 

Subject area	*Electrochemistry*
More specific subject area	*Alkaline electrolysis*
Type of data	*Figure, Tables*
How data was acquired	*XRD (D/max 2500 PC, Rigaku); EDS (X-MAX 50, Horiba Scientific)*
Data format	*Analyzed*
Experimental factors	*Samples were treated in alkaline solution for 24 h at 80 °C to leach Al. Heat treatment of the electrode and powder was carried out at 610 °C for 1 h under hydrogen atmosphere.*
Experimental features	*XRD pattern of samples; chemical composition of samples via EDS*
Data source location	*Daejeon, Korea*
Data accessibility	*Data is displayed within this article.*
Related research article	(Kim et al., 2018) [1]

**Value of the data**

• The data show that the composition of Ni–Al alloy powder is changed when its irradiated by plasma spraying method with a high-temperature environment.
• The data presented in this article provide information on the effects of hydrogen annealing on the Ni–Al alloy powder and Ni–Al alloy electrode coating prepared by APS.
• The data allow other researchers to compare the Raney nickel electrode prepared by APS with those prepared by other methods.

## 1 Data

**Fig. 1:**
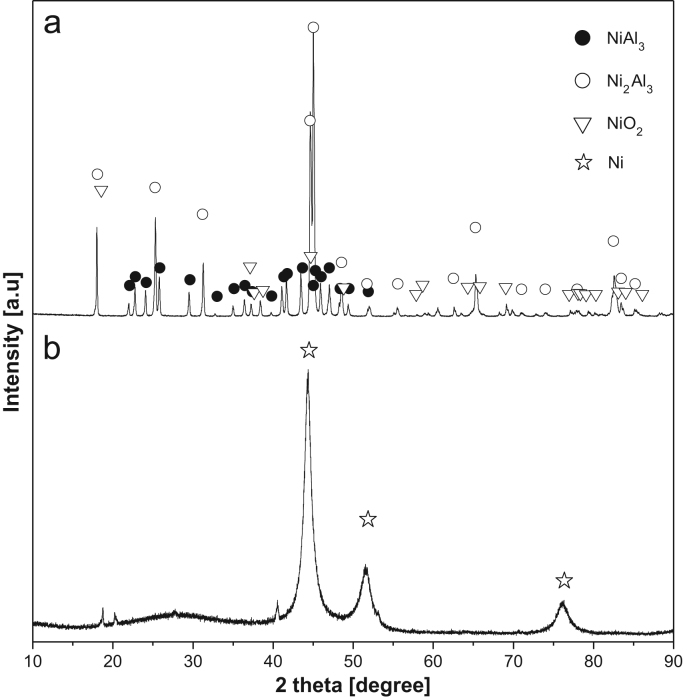
XRD patterns of (a) Ni-Al alloy powder and (b) Raney Ni powder.

Fig. 1 shows the XRD patterns of the Ni-Al alloy powder and Raney Ni powder prepared by dealloying Ni-Al of Al. Table 1 presents the EDS data for the electrode surface coated with Ni-Al alloy by APS and the electrode surface after Al leaching. Table 2 shows the EDS data for the Ni-Al alloy powder and Raney Ni powder prepared by leaching Al from the Ni-Al alloy. Finally, Table 3 shows the EDS data for the Ni-Al-alloy-coated electrode after annealing under hydrogen atmosphere and the Al-leached electrode*.*

**Table 1:** EDX analysis of Ni-Al alloy coated electrode surface (a) as prepared and (b) after Al leaching.

**wt. %**	**As prepared**	**After Al leaching**
**Ni**	53.09	66.25
**Al**	32.65	21.02
**O**	14.26	12.73
**Ni/Al**	1.62	3.15

**Table 2:** EDX analysis of Ni-Al alloy power and Raney Ni powder after Al leaching.

**wt. %**	**Ni-Al alloy powder**	**Raney Ni powder**
**Ni**	60.86	65.54
**Al**	35.21	8.07
**O**	3.92	26.39
**Ni/Al**	1.73	8.12

**Table 3:** EDX analysis of Ni-Al alloy coated electrode surface (a) as prepared with hydrogen heat treatment and (b) after Al leaching.

**wt. %**	**As prepared**	**After Al leaching**
**Ni**	58.06	65.92
**Al**	31.18	9.55
**O**	10.76	6.9
**Ni/Al**	1.86	6.90

## 2 Experimental, materials, and methods

Ni-Al alloy powder (Yakuri Pure Chemicals) was used for XRD and EDS analyses. The powder was sieved to particle sizes of 12–45 μm. Raney Ni powder was prepared by Al leaching. Al was selectively leached for 24 h at 80 °C using 30 wt% potassium hydroxide (Samchun Chemicals, 95.0%) and 10 wt% potassium sodium tartrate tetrahydrate (Alfa Aesar, 99%). After Al leaching, the electrodes were rinsed with distilled water and dried under 1.6 vol% oxygen atmosphere to stabilize the pyrophoric Raney Ni surface. Heat treatment of the coated electrodes under hydrogen atmosphere was conducted by first placing the electrodes in a furnace (WiseTherm), which had been evacuated to create a vacuum and subsequently filled with Ar. Ar was then replaced with H_2_ to 100 vol%, and the electrodes were heat treated for 1 h up to 610 °C at a heating rate of 5 °C/min. After heat treatment, H_2_ was again replaced with Ar and the furnace was allowed to cool down naturally. To prepare the Ni-Al-alloy-coated electrode, a disc-shaped Ni plate with a diameter of about 20 mm and thickness of about 0.6 mm was used for the substrate. Prior to coating, sand blasting was conducted to create roughness. The coating layer was prepared using an APS gun (SG-100, Praxair Surface Technologies) with an output of 600 A, while maintaining a distance of 100 between the spray gun and substrate. During the coating process, the carrier gas, Ar, was supplied at 50 psi. Al leaching and heat treatment under H_2_ atmosphere was conducted in the same manner as that for the Ni-Al alloy powder. The crystalline structures of the Ni-Al alloy and Raney Ni powders were analyzed using an X-ray diffractometer (D/max 2500 PC, Rigaku). XRD measurements were made within the 2θ range of 10–90°, using Cu Kα (λ = 1.5056 Å) radiation energy with a voltage of 40 kV and current of 150 mA. The compositions of the powder and electrode surface were analyzed using an energy dispersive X-ray spectrometer (X-MAX 50, Horiba Scientific).
