# Supplementary material for: Data on the characterization of Raney nickel powder and Raney-nickel-coated electrodes prepared by atmospheric plasma spraying for alkaline water electrolysis
Source: Data Brief. 2018 Nov 3;21:2059–62. doi: 10.1016/j.dib.2018.10.167 (PMC6262154; doi:10.1016/j.dib.2018.10.167)
Supplement: Supplementary file 1 — Supplementary material [file mmc1.doc]

Conflict of Interest and Authorship Conformation Form

Please check the following as appropriate:

- All authors have participated in (a) conception and design, or analysis and interpretation of the data; (b) drafting the article or revising it critically for important intellectual content; and (c) approval of the final version.
- This manuscript has not been submitted to, nor is under review at, another journal or other publishing venue.
- The authors have no affiliation with any organization with a direct or indirect financial interest in the subject matter discussed in the manuscript
- The following authors have affiliations with organizations with direct or indirect financial interest in the subject matter discussed in the manuscript:

Author’s name Affiliation

Ji-Eun Kim Korea Institute of Energy Research, Korea University

Ki-Kwang Bae Korea Institute of Energy Research

Chu-Chik Park Korea Institute of Energy Research

Seong-Uk Jeong Korea Institute of Energy Research

Kyeong-Ho Baik Chungnam National University

Jeong-Won Kim Korea Institute of Energy Research

Young-Ho Kim Chungnam National University

Kyoung-Soo Kang Korea Institute of Energy Research

Ki-Bong Lee Korea University
